# Alterations of Serum Metabolites and Fecal Microbiota Involved in Ewe Follicular Cyst

**DOI:** 10.3389/fmicb.2021.675480

**Published:** 2021-05-12

**Authors:** Tao Feng, Hongxiang Ding, Jing Wang, Wei Xu, Yan Liu, Ákos Kenéz

**Affiliations:** ^1^Institute of Animal Husbandry and Veterinary Medicine (IAHVM), Beijing Academy of Agriculture and Forestry Sciences (BAAFS), Beijing, China; ^2^Joint Laboratory of Animal Science Between IAHVM of BAAFS and Division of Agricultural Science and Natural Resource, Oklahoma State University, Beijing, China; ^3^College of Animal Science and Technology, Henan University of Science and Technology, Luoyang, China; ^4^College of Animal Science and Technology, Hebei North University, Zhangjiakou, China; ^5^Department of Infectious Diseases and Public Health, City University of Hong Kong, Hong Kong, China

**Keywords:** sheep, follicular cysts, microbial diversity, metabolome, host-microbiota interactions

## Abstract

While the interactions of the gut microbiome and blood metabolome have been widely studied in polycystic ovary disease in women, follicular cysts of ewes have been scarcely investigated using these methods. In this study, the fecal microbiome and serum metabolome were used to compare between ewes diagnosed with ovarian cystic follicles and ewes with normal follicles, to investigate alterations of the fecal bacterial community composition and metabolic parameters in relation to follicular cystogenesis. Ewes from the same feeding and management system were diagnosed with a follicular cyst (*n* = 6) or confirmed to have normal follicles (*n* = 6) by using a B-mode ultrasound scanner. Blood serum and fresh fecal samples of all ewes were collected and analyzed. The α-diversity of fecal microbiome did not differ significantly between follicular cyst ewes and normal follicle ewes. Three genera (*Bacteroides*, *Anaerosporobacter*, and *Angelakisella*) were identified and their balance differentiated between follicular cyst and normal follicle ewes. Alterations of several serum metabolite concentrations, belonging to lipids and lipid-like molecules, organic acids and derivatives, organic oxygen compounds, benzenoids, phenylpropanoids and polyketides, and organoheterocyclic compounds, were associated with the presence of a follicular cyst. Correlation analysis between fecal bacterial communities and serum metabolites indicated a positive correlation between *Anaerosporobacter* and several fatty acids, and a negative correlation between *Bacteroides* and L-proline. These observations provide new insights for the complex interactions of the gut microbiota and the host serum lipid profiles, and support gut microbiota as a potential strategy to treat and prevent follicular cysts in sheep.

## Introduction

Cystic ovarian follicle is one of the ovarian dysfunctions in humans and livestock, resulting mainly from several alterations in follicle development and ovulatory mechanisms, causing female infertility (Mutinati et al., 2013; Ortega et al., 2016). It is generally recognized that functional alterations of the hypothalamus-pituitary-gonadal axis caused by imbalance of ovarian endocrine homeostasis is the main cause of follicular cysts (Christman et al., 2000; Medan et al., 2004; Mutinati et al., 2013). Absence or abnormal release of hypothalamic gonadotropin-releasing hormone (GnRH) or lack of luteinizing hormone (LH) surge were considered as one of the endocrine reasons to induce a follicular cyst, identified as a target for symptomatic therapies to treat infertility (Medan et al., 2004; Abdalla et al., 2020). In humans, ovarian diseases with a phenotype of follicular cysts are collectively referred to as polycystic ovary syndrome (PCOS). For the past few years, the relationship between the gut microbiota and metabolic status attracted significant attention to reveal the etiology and pathological mechanisms of PCOS, based on intestinal bacterial communities influencing energy absorption, short chain fatty acid production, and lipopolysaccharide release (Liu et al., 2017; Zhao et al., 2020). Previous studies also showed that bacterial diversity of gut microbial communities had an effect on PCOS depending on host metabolic parameters (Lüll et al., 2020). Additionally, altered fecal microbiome and metabolome and their associations with diseases, such as kidney disease and chronic obstructive pulmonary disease, were reported in humans (Chen et al., 2019; Bowerman et al., 2020). Further associations were revealed between cysteine levels on pregnancy outcome in sows and myostatin phenotype affecting lean meat proportion in pigs (Ding et al., 2019; Pei et al., 2021).

Ovarian cyst is one of the reasons of infertility in sheep and goat (Medan et al., 2004; Palmieri et al., 2011). The incidence of follicular cysts in sheep was reported highly variable, ranging from 0.2 to 6% (Smith et al., 1999; Palmieri et al., 2011). According to the previous studies, the reason of ewes’ follicular cysts includes inhibition of preovulatory LH surge by adrenocorticotrophic hormone (ATCH) (Palmieri et al., 2011), lower concentration of plasma progesterone (Medan et al., 2004), overweight (Christman et al., 2000), and *Toxoplasma gondii* infection (Moraes et al., 2010). As far as we know, there were few studies about the relationship between the gut microbiota or blood metabolome and follicular cysts in ewes, and about the associations of the gut microbiota and the blood metabolome in relation to cystogenesis in sheep. Thus, the aims of the present study were: (1) to characterize the alterations of fecal microbial communities, (2) to identify patterns of serum metabolome profiles, and (3) to reveal the associations of gut microbiota and metabolome in follicular cysts ewes compared with normal follicle ewes. The objective of this research was to provide potential targets of gut microbiota or metabolic pathways for therapeutic and preventive interventions of follicular cyst in sheep.

## Materials and Methods

### Experimental Station

Experiments were performed at the Experimental Station of Beijing Academy of Agriculture and Forestry Sciences in Yangyuan County, Zhangjiakou City, Hebei Province, Northeast of China. All the experiments were carried out according to the International Guiding Principles for Biomedical Research Involving Animals, and the respective permit was granted by Beijing Academy of Agriculture and Forestry Sciences (SYXQ-2012-0034).

### Ewes and Reproduction Management

A total of 320 crossbreed ewes (*Ovis aries*) of Merino rams and Small Tailed Han ewes aged 2–4 years were housed in a four sheltered outdoor paddocks and were fed a total mixed ration (TMR) of 2,000 g per head per day after weaning. The TMR composition was based on the recommendations of sheep feeding standards in China (NY/T816-2004). Clean water and mineralized salt licks were available *ad libitum*. From 1 May to 31 May, eight rams were put into each ewe paddock to mate ewes naturally. On 5 July, conception was identified by pregnancy diagnosis using a B-mode ultrasound scanner (Honda HS-1600V, Honda Electronics, Tokyo, Japan).

### Ultrasonography

Besides pregnancy diagnosis, the B-mode scanner was used to diagnose ovarian follicular cyst by transrectal ultrasonography collaborated with a 7.5 MHz transducer as mentioned by Steckler et al. (2008) and Palmieri et al. (2011). Based on the follicular diameter and the presence or absence of a fetal sac, ewes were divided into three groups: pregnant, follicular cyst, and non-pregnant with normal follicles. Ewes were diagnosed with follicular cyst when the follicle diameter was greater than 10 mm (Medan et al., 2004; Palmieri et al., 2011). Ewes diagnosed with ovarian follicular cyst were re-evaluated after 8 days, and animals were enrolled for sample collection if the follicular cyst was confirmed to still be greater than 10 mm.

### Fecal Sample Collection and Microbiota Analysis

Fresh fecal samples were individually collected from six ewes with a follicular cyst diameter greater than 10 mm, after the second ultrasonography. Fresh fecal samples of six non-pregnant ewes with normal follicles from the same herd were collected at the same time. The samples were quickly frozen in liquid nitrogen and submitted to the laboratory. Genomic DNA of the 12 samples were extracted using an E.Z.N.A. Stool DNA Isolation Kit (Omega Bio-tek, Norcross, GA, United States) following the recommended instructions and confirmed with 1.2% agarose gel, of which the DNA yield and purity were measurement of absorbance using NanoDrop 2000 UV-vis spectrophotometer (Thermo Fisher Scientific, Wilmington, United States). A pair of barcode-modified universal primer 338F and 806R (forward: 5′-ACTCCTACGGGAGGCAGCA-3′; reverse: 5′-GGACTACHVGGGTWTCTAAT-3′) was used to amplify the V3 + V4 hypervariable fragments from the bacterial 16S rDNA (Wang et al., 2018). The PCR products were purified using an AxyPrep DNA Gel Extraction Kit (Axygen Biosciences, Union City, CA, United States), following the manufacturer’s instructions. The DNA fragment amplicons were sequenced on an Illumina MiSeq PE300 platform/NovaSeq PE250 platform (Illumina, San Diego, United States) according to the standard procedures of Majorbio Bio-Pharm Technology Co. Ltd. (Shanghai, China). The raw reads were approved by the NCBI Sequence Read Archive (SRA) database with accession number SRP308293.

The raw sequencing reads of 16S rRNA gene were demultiplexed, quality-filtered by fastp version 0.20.0 (Chen et al., 2018) and merged by FLASH version 1.2.7 (Magoč and Salzberg, 2011). Optimized, high-quality sequences were clustered using UPARSE version 7.1 into operational taxonomic units (OTUs) at 97% sequence identity, and chimeric sequences were discerned and filtered out. The taxonomy of each OTU representative sequence was analyzed by Ribosomal Database Project (RDP) naive Bayesian classifier against the 16S rRNA database (Release 138)^1^ (Wang et al., 2018). Alpha diversity (Shannon and Simpson estimators for diversity evaluation, Chao and ACE estimators for abundance evaluation) was analyzed by Mothur v. 1.31.2. Principle coordinates analysis (PCoA) was used to visualize differences in fecal community composition reflecting its beta diversity. The linear discriminant analysis effect size (LEfSe) algorithm was performed to identify the taxa differences responsible for different groups. The biomarkers of LEfSe analysis conducted in the microbiota study had an effect-size threshold of two. PICRUSt2 was used to identify metabolic activities of the gut microbiota (Zhang et al., 2021). Predicted metabolic profile for Kyoto Encyclopedia of Genes and Genomes (KEGG) Orthologs (KO) were mapped on database matching^2^.

### Blood Sample Collection and Metabolomics Analysis

Blood was collected from the same ewes enrolled for fecal microbiome analysis (*n* = 6 with follicle cyst, *n* = 6 with normal follicles) via jugular venipuncture into 10-mL vacuum tubes. Blood samples were undisturbed and kept at room temperature for 4 h and then centrifuged at 2,000 *g* for 30 min at 4°C to isolate the sera, which were subsequently stored at −80°C until further analysis.

Using 100 μL serum, metabolites were extracted using methanol. Extracts were sonicated, and after centrifugation, the supernatants were gently added to sample vials for LC-MS/MS analysis. A pooled quality control sample (QC) was used for system conditioning and quality control. Chromatographic separation of the metabolites was operated on a Thermo UHPLC system equipped with an ACQUITY UPLC HSS T3 (100 mm × 2.1 mm i.d., 1.8 μm; Waters, Milford, United States). Following LC-MS/MS analyses, the raw data were inputted into the Progenesis QI 2.3 (Non-linear Dynamics, Waters, United States) for peak picking and alignment. Mass spectra of these metabolic characteristics were discerned through the accurate mass, MS/MS fragments spectra, and isotope ratio difference, by scanning in publicly available biochemical databases such as the Human metabolome database (HMDB)^3^ and Metlin database^4^. A multivariate statistical analysis was conducted using ‘‘ropls’’ (Version1.6.2)^5^ R package from Bioconductor on Majorbio Cloud Platform^6^. Principle component analysis (PCA) was applied to check outliers and present trends. Partial least squares-discriminant analysis (PLS-DA) was used to identify the general metabolic changes in serum of sheep with or without follicular cyst. Variable importance in the projection (VIP) was computed by an orthogonal partial least squares discriminant analysis (OPLS-DA) model. Differential metabolites between groups were identified (*P* < 0.05, VIP-value > 1), and annotated into their biochemical pathways through metabolic enrichment and pathway analysis based on database matching (KEGG) (see text footnote 2). Further, Volcano plot was used to compare the size of the fold change to statistical significance. Regarding VIP value of metabolites, ^∗^ means significant difference between ewes with follicular cyst and normal follicle (*P* < 0.05), ^∗∗^ means significant difference (*P* < 0.01), and ^∗∗∗^ means significant difference (*P* < 0.001).

### Correlation Between Serum Metabolites and Fecal Microbial Taxa

The cooperativity of two-dimensional shapes produced from superimposition of PCA from microbiome and metabolome was conducted by Procrustes analysis (PA). Mainly, the correlation between relative abundance of fecal microbiota at genus levels and differential metabolites was analyzed by R package ggplot2 (McHardy et al., 2013; Mazhar et al., 2021). *P* <0.05 were considered to have significant difference. Representation of the *P*-value is as follows: ^∗^ *P* < 0.05, ^∗∗^ *P* < 0.01, and ^∗∗∗^ *P* < 0.001.

## Results

### Cysts Diagnosis

**FIGURE 1:**
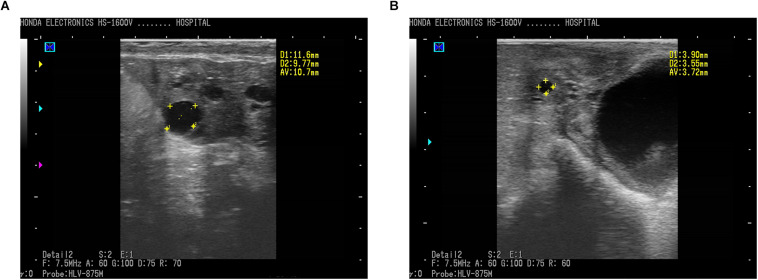
Ultrasound photos of ovaries of ewes imaged using a 7.5 MHz transducer and B-mode scanner. **(A)** Follicular cyst with 10.70 mm in diameter in sheep. **(B)** Normal follicle with 3.72 mm in diameter. Scale bars represent 10 mm. Follicles (round black structures) marked with yellow crosses showed anechoic structure.

In the present study, transrectal ultrasonography by a B-mode scanner identified ovarian follicular cysts clearly and effectively in sheep. Ewes diagnosed with a follicular cyst demonstrated a sharp image of anechoic (round and black) structure with an antrum greater than 10 mm in diameter (Figure 1A), while sheep with normal follicles showed a smaller antrum (Figure 1B). The average diameter of the follicles in cyst and normal sheep were 11.6 ± 0.5 mm and 3.6 ± 0.3 mm, respectively.

### Fecal 16S rRNA Sequencing

A total of 545,894 reads were sequenced in the amplified 16S rRNA genes, after quality checks in 12 samples. As for follicular cyst ewes, the mean (SD in parentheses) reads was 46,627 (2,407), while 44,355 (4,980) reads were obtained in normal follicle ewes. Among the high-quality sequences, the minimum length was 248 bp and the maximum length was 511 bp. The read length for all samples was 413 bp on average, in which more than 99.9% of reads exceeded 400 bp. Reads were clustered into 2,000 OTUs using a 97% similarity threshold. OTUs ranged from 346 to 778 per sample were obtained. Based on sequencing results and rarefaction analysis, the depth of sequence obtained was adequate to reflect species richness, indicating that the sequencing system (Illumina Miseq) we used identified most of the fecal bacterial diversity in the present study.

### Alpha Diversity of Fecal Microbiota

**FIGURE 2:**
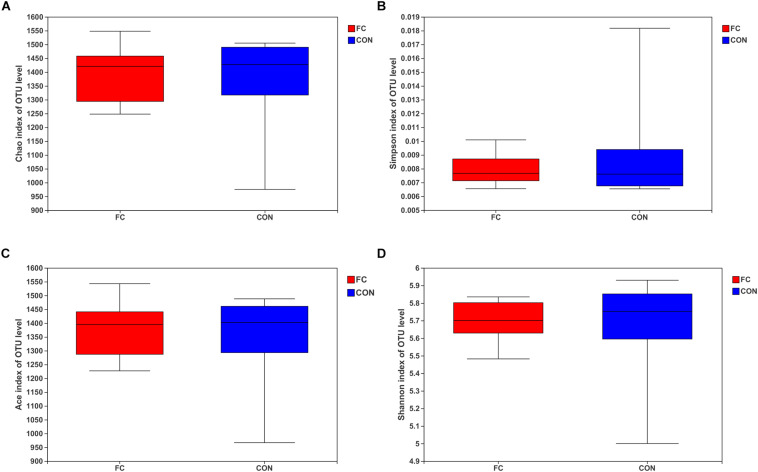
Alpha diversity for the fecal microbiota in follicular cyst and normal follicle ewes. **(A)** Chao index; **(B)** Simpson index; **(C)** ACE index; **(D)** Shannon index.

The Chao, Simpson, ACE, and Shannon estimators were used to evaluate fecal microbiome taxon abundance and diversity (Figure 2). No significant difference was found in the fecal microbiome diversity comparing the follicular cyst ewes with the normal follicle ewes by student’s *t*-test (all *P* > 0.05). Meanwhile, the Good’s coverage estimator was more than 99% for fecal samples of follicular cyst ewes and normal follicle ewes, indicating that the dominant bacterial phenotypes were included in our study.

### Fecal Microbiota Composition

A total of 2,000 OTUs were identified in fecal samples of follicular cyst ewes and normal follicle ewes, in which 1,645 OTUs were co-existent, 195 OTUs were follicular cyst ewes only, and 160 OTUs were normal follicle ewes only.

**FIGURE 3:**
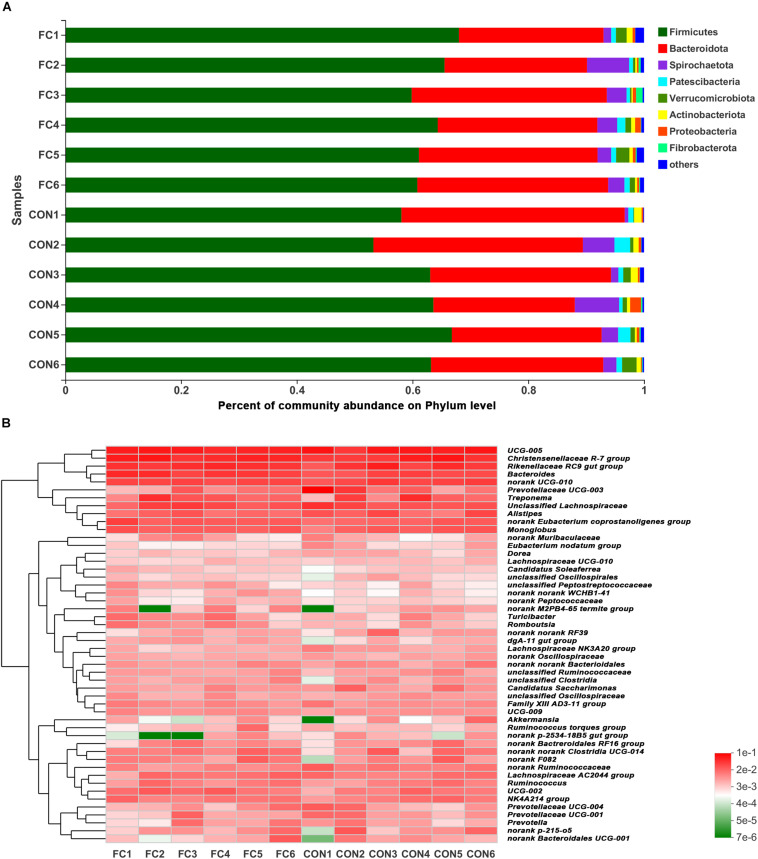
Phylum-level and genus-level fecal microbiota profiles in follicular cyst ewes and normal follicle ewes. **(A)** Stacked column chart showing the relative phylum-level bacterial abundance (>1%) per fecal sample. **(B)** The heatmap shows the relative genera-level bacterial abundance. FC represents ewes with follicular cysts and CON represents ewes with normal follicle. Numbers represent individual animals.

At phylum level, Firmicutes and Bacteroidetes (Firmicutes > Bacteroidetes) were the two dominant taxa in both groups and accounted for 92.41% for follicular cyst ewes and 92.35% for normal follicle ewes of total phylum, on average. Other three phyla were present at lower frequencies, including Spirochaetota, Patescibateria, and Verrucomicrobiota. In fecal samples of follicular cyst ewes, these three taxa (Spirochaetota > Verrucomicrobiota > Patescibateria) accounted for 5.42% on average, while in normal follicle ewes they (Spirochaetota > Patescibateria > Verrucomicrobiota) accounted for 5.70% on average (Figure 3A).

At genus level, a hierarchically clustered heatmap of the fecal microbiota composition of ewes was shown in Figure 3B. *UCG-005* (10.37%), *Christensenellaceae_R-7_group* (7.48%), *Rikenellaceae_RC9_gut_group* (6.20%), *Prevotellaceae_UCG-003* (5.18%), and *Bacteroides* (4.05) were the top five dominant genera in follicular cyst ewes on average, while *UCG-005* (9.64%), *Christensenellaceae_R-7_group* (8.81%), *Rikenellaceae_RC9_gut_group* (6.78%), *Bacteroides* (6.26%), and *Treponema* (3.30%) were the top five dominant genera in normal follicle ewes on average.

**FIGURE 4:**
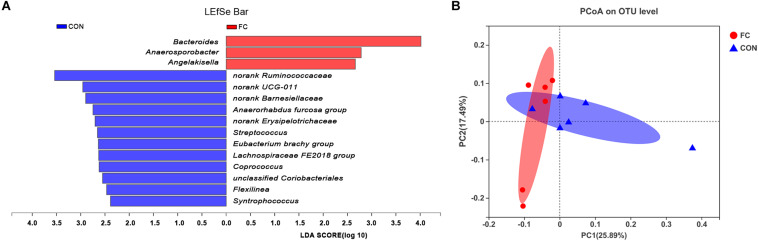
Differences between bacterial taxa in follicular cyst ewes and normal follicle ewes. **(A)** The linear discriminant analysis (LDA) effect size (LEfSe) algorithm displayed significant differences between follicular cyst ewes and normal follicle ewes at genus level. **(B)** The principal coordinate analysis (PCoA) plots of the fecal microbiota based on the unweighted UniFrac showed the extent of variation in fecal microbial community between follicular cyst ewes and normal follicle ewes at OUT level. FC represents ewes with follicular cysts and CON represents ewes with normal follicle. Red bars represent OTU that are more abundant in follicular cyst ewes’ fecal samples than in normal follicle controls.

LEfSe analysis manifested significant differences between follicular cyst ewes and normal follicle ewes from phylum to genus level according to relative OTU abundance (Supplementary Figure 1). Ewes with follicular cyst had enriched Bacteroidaceae at family level, *Bacteroides*, *Anaerosporobacter*, and *Angelakisella* at genus level. However, ewes with normal follicle were abundant with 1 at phylum level, 1 at class level, 3 at order level, 5 at family level, and 12 at genus level (Figure 4A and Supplementary Figure 1).

Principle coordinates analysis (PCoA) indicated differences of fecal bacterial communities between ewes with follicular cyst and normal follicle (Figure 4B).

A total of 204 functional pathways were predicted with PICRUSt2 by comparing against KEGG orthologs. KEGG pathways including ABC transporters, purine metabolism and aminoacyl-tRNA biosynthesis were higher in follicular cyst ewes than normal follicle ewes, which showed a good response to KEGG pathways enrichment in metabolomics.

### Serum Metabolite Profile

**FIGURE 5:**
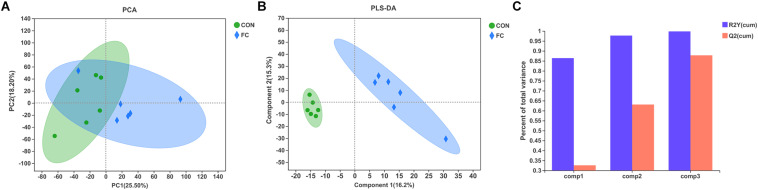
Difference of serum metabolite profiles of ewes with follicular cyst or normal follicle. **(A)** PCA score plot for serum metabolites of ewes with follicular cyst or normal follicle. **(B)** PLS-DA score plot for serum metabolites of ewes with follicular cyst or normal follicle. **(C)** The model overview showing high R2Y and Q2 in PLS-DA. The values of R2Y and Q2 were 0.998 and 0.878, respectively. FC represents ewes with follicular cysts and CON represents ewes with normal follicle.

In the serum LC-MS spectra of ewes with follicular cyst or with normal follicle, 10,598 metabolites were initially found. After quality control and discernment, 948 compounds were reliably detected. The PCA score plot presented that the first and second principal components (PCs) covered 25.50% and 18.20% of the variation, respectively (Figure 5A). Scores representing follicular cyst and normal follicle samples were separated in the PCA plot. PLS-DA was performed to analyze the serum metabolome profile variations between the follicular cyst and normal follicle ewes. As shown in Figure 5B, the PLS-DA analysis demonstrated that the serum metabolites of the follicular cyst ewes distinctly differed from those of the normal follicle ewes. Correspondingly, the values of R2Y and Q2 were 0.998 and 0.878, respectively (Figure 5C), indicating good interpretability and predictability by this PLS-DA model. A value of Q2 = 1 indicates a perfect discrimination of metabolite profiles between groups.

### Difference in Serum Metabolite

**FIGURE 6:**
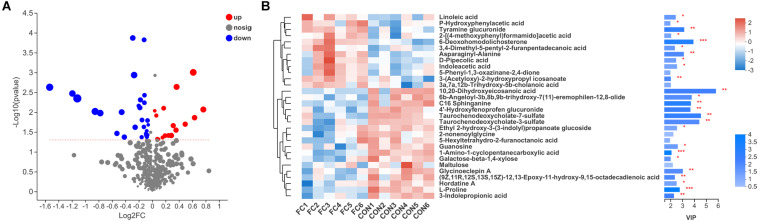
Serum metabolite profiles of ewes with follicular cyst or normal follicle. **(A)** Enhanced Volcano plots of OPLS-DA showing fold changes (log_2_FC) and the negative logarithm (base 10) of the *P*-values of 44 differential serum metabolites in follicular cyst ewes compared with normal follicle ewes. **(B)** Expression profile, VIP score, and *P-*value of the top 30 differential serum metabolites in follicular cyst ewes compared with normal follicle ewes. *Significant difference in serum metabolome between ewes with follicular cyst and normal follicle (*P* < 0.05), **significant difference (*P* < 0.01), and ***significant difference (*P* < 0.001).

In comparison, a total of 44 differential serum metabolites were found according to the Volcano plot, of which 16 metabolites showed up-regulation and 28 showed down-regulation in ewes with follicle cyst (Figure 6A and Supplementary Table 1). A total of 40 differential serum metabolites were annotated in 7 superclasses according to HMDB database, of which 20 belonged to lipids and lipid-like molecules, 7 belonged to organic acids and derivatives, 4 belonged to organic oxygen compounds, 3 belonged to benzenoids, 3 belonged to phenylpropanoids and polyketides, 2 belonged to nucleosides, nucleotides, and analogs, and 1 belonged to organoheterocyclic compounds. Expression profile and VIP of top 30 metabolites based on the OPLS-DA model was shown in Figure 6B.

Regarding KEGG pathway, the four pathways including at least two differential serum metabolites annotated were aminoacyl-tRNA biosynthesis (L-proline and L-histidine), protein digestion and absorption (L-proline and L-histidine), purine metabolism (Diadenosine tetraphosphate and Guanosine), and ABC transporters (L-proline and L-histidine), respectively. Based on our results, the serum metabolome profiles of ewes were affected by the presence or absence of follicular cyst.

### Correlation Between Serum Metabolites and Fecal Microbial Taxa

**FIGURE 7:**
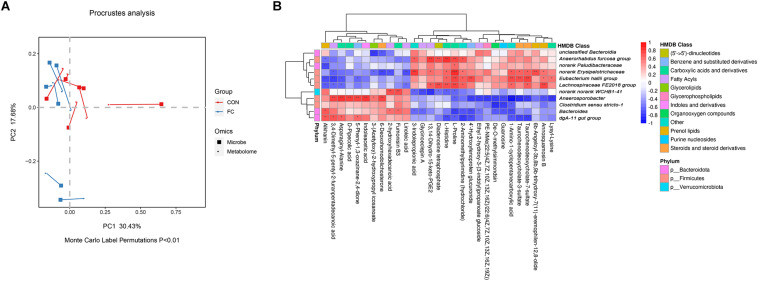
Correlation between serum metabolites and fecal microbial taxa in ewes. **(A)** Procrustes analysis of fecal microbiome and serum metabolome in ewes with follicular cyst and normal follicle. Fecal and serum samples are showed as squares and dots, respectively. Fecal and serum samples from the same individual are connected by red (control ewes) and blue (follicular cyst ewes) lines. **(B)** Correlation analysis between fecal microbiome and serum metabolome in the ewes with follicular cyst and normal follicle. Red represents a positive correlation, while blue represents a negative correlation. *Significant correlation between fecal microbiome and serum metabolome (*P* < 0.05), **significant correlation (*P* < 0.01), and ***significant correlation (*P* < 0.001).

In order to identify if there was any inter-omic syntropy, a two-dimensional principal component distribution plot (30.43% in PC1 and 17.68% in PC2) was generated with square (microbe) or dot (metabolome) (Figure 7A). Procrustes analysis showed a strong cooperativity of fecal microbiome profiles and serum metabolome (Figure 7A: Monte Carlo *P* < 0.01). To further investigate the relationship between metabolites and microbes, a correlation matrix was conducted based on the Pearson’s correlation coefficient (Figure 7B). A total of 11 genera of microorganisms and 29 metabolites were included in the heatmap matrix. The results demonstrated several significant metabolite–microbe relationships, such as L-proline had a strong positive correlation (*P* < 0.05) with *Anaerorhabdus* but had a strong negative correlation (*P* < 0.01) with *Bacteroides*.

## Discussion

### Alteration of Fecal Microbiota in Ewes With Follicular Cyst or Normal Follicle

Polycystic ovary syndrome (PCOS) is a common ovarian disease in women with a prevalence of 8–13% (Zhao et al., 2020). Multiple studies have confirmed the close relationship between the gut microbiota and PCOS (Mammadova et al., 2020; Zhao et al., 2020). Follicular cysts were also discovered in sheep (Christman et al., 2000; Palmieri et al., 2011) and goat (Medan et al., 2004; Maia et al., 2018). To our knowledge, there has been no scientific report on the correlation between gut microbiota and follicular cyst in sheep. Several studies were reviewed that PCOS patients had a decreased α-diversity and different β-diversity composition in gut microbiota compared with healthy controls (Guo et al., 2021). In the present study, no significant change was identified for α-diversity index of OTU level in fecal microbiota of follicular cyst ewes compared with normal follicle ewes. As for β-diversity composition in fecal microbial community, ewes with a follicular cyst were enriched at family level (Bacteroidaceae) and at genus level (*Bacteroides*, *Anaerosporobacter*, and *Angelakisella*). Bacteroidaceae was reported with a lower percent of relative abundance in gastrointestinal microbiota of PCOS girls (Jobira et al., 2020), while the family Bacteroidaceae had a higher level either in gut microbial community of women phenotyped as PCOS with insulin resistance or PCOS alone (Zeng et al., 2019). At genus level, bacterial taxa including *Coprococcus*, *Bacteroides*, *Prevotella*, *Lactobacillus*, *Parabacteroides*, *Escherichia/Shigella*, and *Faecalibacterium prausnitzii* were reviewed to be clearly altered in the gut microbiota of PCOS patients (Guo et al., 2021), of which *Bacteroides* was the most significant alteration in fecal microbiota of follicular cyst ewes in our study. Up to date, there has been no report on the relationship between fecal *Anaerosporobacter* genus or *Angelakisella* genus and follicular cysts. It was speculated that *Anaerosporobacter* may cause vascular damage and worsen renal function in murine models (Li et al., 2020), and the alteration in abundance of *Anaerosporobacter* in gut microbiome composition may cause coronary artery diseases (Toya et al., 2020). *Angelakisella*, a new bacterial species isolated from human ileum (Mailhe et al., 2017), was identified to regulate short chain fatty acids production in gut microbiota (Qiu et al., 2021). Alterations of *Anaerosporobacter* genus and *Angelakisella* genus in gut microbiota are firstly reported to have potential effects on follicular cyst in sheep, however, the precise mechanism of the two bacterial taxa in cyst development and cyst maintenance warrants further studies.

### Alterations of Serum Metabolites in Ewes With Follicular Cyst or Normal Follicle

The serum metabolome profile of ewes with follicular cyst or normal follicle were found to be different using LC-MS/MS metabolomics analysis in our study. Alterations of serum compounds belonging to lipids and lipid-like molecules, organic acids and derivatives, organic oxygen compounds, benzenoids, phenylpropanoids and polyketides, and organoheterocyclic compounds were highlighted between follicular cyst and normal follicle ewes. Furthermore, according to the OPLS-DA model and VIP values, several metabolites are suggested as potential biomarkers or key metabolites to indicate the metabolic basis of follicular cysts development and maintenance in sheep.

PCOS was considered to be associated with turbulence of lipid metabolism in females (RoyChoudhury et al., 2016). As shown in Figure 6B and Supplementary Table 1, compounds annotated to lipids and lipid-like molecules were the most dominantly altered metabolites in follicular cyst ewes, according to HMDB database classification in our study. Linoleic acid (C18:2, n-6) is a common fatty acid in plasma and in granulosa cells in sheep, which is an essential fatty acid for arachidonic acid (C20:4, n-6) and eicosanoids synthesis (Mattos et al., 2000; Wonnacott et al., 2010). Linoleic acid inhibited oocyte maturation in cattle both *in vivo* and *in vitro* (Homa and Brown, 1992; Marei et al., 2010). However, in sheep, linoleic acid had an inhibitory effect on embryo development *in vitro* (Amini et al., 2016).

3,4-dimethyl-5-pentyl-2-furannonanoic acid, furan fatty acids with pentyl side chain, was produced from linoleic acid (Batna et al., 1993), and had a capacity to confront the intracellular negative effects consequences resulting from oxidative stress (Teixeira et al., 2013). The effect of 3,4-dimethyl-5-pentyl-2-furannonanoic acid on follicular cyst in sheep is still unknown and requires further studies.

Amino acids and their metabolic intermediates are of huge influence on anabolism and metabolic pathways. A disequilibrium of normal amino acid levels could trigger pathophysiological changes causing of infertility (Wu, 2009). In PCOS patients, serum levels of two amino acids, including proline and histidine, were reported to be down-regulated, compared with healthy controls (Atiomo and Daykin, 2012; Unni et al., 2015; RoyChoudhury et al., 2016). Proline and histidine were demonstrated to have negative association with inflammation and oxidative stress (Niu et al., 2012). Low levels of proline and histidine in PCOS patients might be a result of an increased utilization of proline and histidine to counteract oxidative stress during follicular cysts (Unni et al., 2015). Therefore, strategies to increase the levels of proline and histidine are anticipated to counteract the disorders caused by inflammation and oxidative stress during follicular development.

Indoleacetic acid, an organoheterocyclic compounds, is a major degradation product of L-tryptophan (an essential amino acids for ruminant animals), found in ruminal bacteria, as well as in blood and in several tissues in sheep and goats (Mohammed et al., 2003; Andrade et al., 2005). In ovarian tissues, indoleacetic acid was suspected to bind to growth factors (Ferreira et al., 2001), consequently improving the enzyme activity of the peroxidases during lipid peroxidation (Candeias et al., 1995). An *in vitro* study showed that lower concentration of indoleacetic acid improved follicle development, while higher doses demonstrated cytotoxicity in the absence of follicle-stimulating hormone (FSH) (Costa et al., 2010). In the present study, high abundance of indoleacetic acid likely had a negative effect on normal follicle development.

### Correlation Between Fecal Bacterial Communities and Serum Metabolites in Ewes

Potential mechanisms of follicular cyst development may include circulating lipid and amino acid levels, affected by gut microbial composition. Little is known about the relationship between *Anaerosporobacter* and fatty acid absorption and metabolism. In the present study, positive correlations were identified between *Anaerosporobacter* and six serum compounds including 6-deoxohomodolichosterone, 3-(acetyloxy)-2-hydroxypropyl icosanoate, 3,4-dimethyl-5-pentyl-2-furanpentadecanoic acid, indoleacetic acid, asparaginyl-alanine, and D-pipecolic acid. Negative correlations were found between *Bacteroides* and L-proline and 3-indolepropionic acid. Changed abundance of *Anaerosporobacter* in fecal bacterial community and fatty acid composition in blood had been proved to associate with artery function (Toya et al., 2020; Samson et al., 2021). Coccidiosis, a disease due to *Eimeria* infection, was characterized by an increased abundance of *Bacteroides* and a decreased serum concentration of histidine and proline in mice (Huang et al., 2018). Alterations of gut microbiota and serum metabolome and their correlations could better explain the formation and maintenance of follicle cysts in ewes. Nevertheless, the causes of sheep follicular cyst were found by statistical analysis of omics data and from a relatively small number of samples. Further studies with evaluation the effects of microbiology or/and metabolites identified in the present study on ewe follicular cyst, and with larger size samples, are desired to validate our findings.

## Conclusion

To conclude, we found correlations between the gut microbiome composition and various circulating metabolites in relation to follicle cyst development in ewes, suggesting complex interactions between gut microbiota, serum metabolome, and ovarian follicle dysfunction. Ewes’ follicular cyst development may be affected by three pathways: (1) a high intestinal abundance of *Bacteroides*, *Anaerosporobacter* and *Angelakisella*; (2) accumulation of organic acids and derivatives (such as D-pipecolic acid, asparaginyl-alanine, fumonisin B3) and lipids and lipid-like molecules (linoleic acid, 2-hydroxyhexadecanoic acid, 3,4-dimethyl-5-pentyl-2-furanpentadecanoic acid) in serum; and (3) a respective interactions of fecal microbiota and serum metabolites. A bacteria-metabolite multilayer can enhance our comprehension of the metabolite pathways significantly associated to the microbial communities of follicular cysts in sheep. Based on the present multi-omics study, further studies are needed to verify the results.

## Data Availability Statement

The datasets presented in this study can be found in online repositories. The names of the repository/repositories and accession number(s) can be found below: https://www.ncbi.nlm.nih.gov/sra/?term=SRP308293.

## Ethics Statement

The animal study was reviewed and approved by the Beijing Academy of Agriculture and Forestry Sciences.

## Author Contributions

TF and YL conceived the study. JW and TF obtained the funding. HD performed the animal trials and the data collection. WX and TF performed the data interpretation. TF and HD wrote the manuscript. WX and ÁK performed the manuscript revision. All authors read and approved the final manuscript content.

## Conflict of Interest

The authors declare that the research was conducted in the absence of any commercial or financial relationships that could be construed as a potential conflict of interest.

## Footnotes

1 http://www.arb-silva.de

2 http://www.genome.jp/kegg/

3 http://www.hmdb.ca/

4 https://metlin.scripps.edu/

5 http://bioconductor.org/packages/release/bioc/html/ropls.html

6 https://cloud.majorbio.com

## Supplementary Material

Supplementary Figure 1 The linear discriminant analysis (LDA) effect size (LEfSe) algorithm of significant differences between fecal bacterial taxa in follicular cyst ewes and normal follicle ewes from family to genus level.

Supplementary Table 1 List of identified metabolites differentially accumulated in serum samples from follicular cyst ewes compared with control ewes.
