# Supplementary figures and images for: Alterations of Serum Metabolites and Fecal Microbiota Involved in Ewe Follicular Cyst
Source: Front Microbiol. 2021 May 12;12:675480. doi: 10.3389/fmicb.2021.675480 (PMC8149755; doi:10.3389/fmicb.2021.675480)

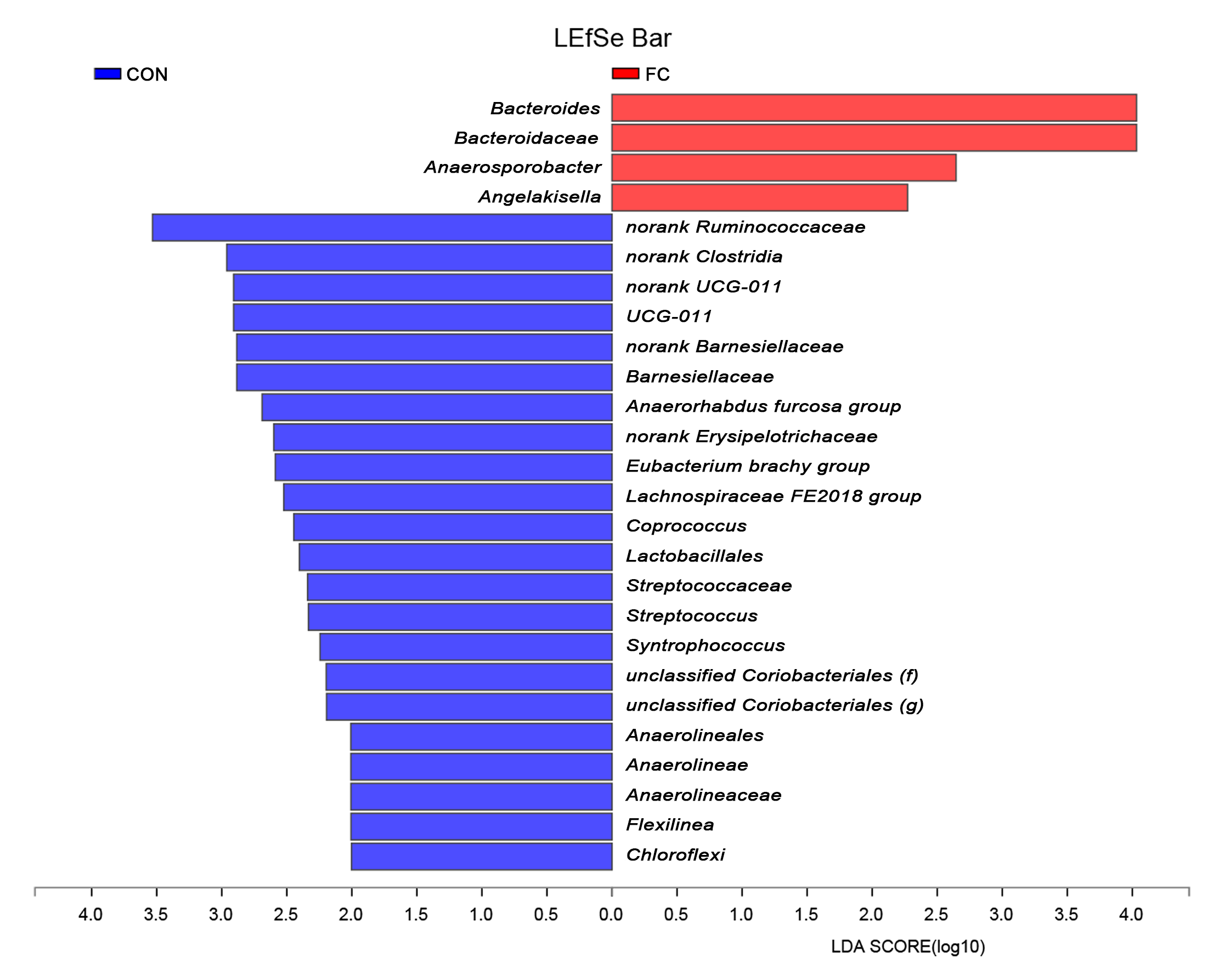

Supplement: Supplementary Figure 1 — The linear discriminant analysis (LDA) effect size (LEfSe) algorithm of significant differences between fecal bacterial taxa in follicular cyst ewes and normal follicle ewes from family to genus level. [file Image_1.TIF]
