# Supplementary material for: Alterations of Serum Metabolites and Fecal Microbiota Involved in Ewe Follicular Cyst
Source: Front Microbiol. 2021 May 12;12:675480. doi: 10.3389/fmicb.2021.675480 (PMC8149755; doi:10.3389/fmicb.2021.675480)
Supplement: Supplementary Table 1 — List of identified metabolites differentially accumulated in serum samples from follicular cyst ewes compared with control ewes. [file Table_1.docx]

Table 1. List of identified metabolites differentially accumulated in serum samples from follicular cyst ewes compared with control ewes

| Metabolite (44) | VIP^1^ | *P* value | FC^2^ | Regulation^3^ |
| --- | --- | --- | --- | --- |
| Lipids and lipid-like molecules (20) | | | | |
| Arachidonoyl Serinol | 2.443 | 0.014 | 1.551 | Up |
| 6-Deoxohomodolichosterone | 3.649 | 0.001 | 1.530 | Up |
| Linoleic acid | 2.248 | 0.020 | 1.408 | Up |
| 3,4-Dimethyl-5-pentyl-2-furanpentadecanoic acid | 2.258 | 0.022 | 1.243 | Up |
| 3-(Acetyloxy)-2-hydroxypropyl icosanoate | 1.871 | 0.008 | 1.126 | Up |
| LysoPC(22:5(4Z,7Z,10Z,13Z,16Z)) | 1.504 | 0.047 | 1.054 | Up |
| 2-hydroxyhexadecanoic acid | 1.463 | 0.012 | 1.043 | Up |
| 13,14-Dihydro-15-keto-PGE2 | 1.856 | 0.016 | 0.953 | Down |
| PE-NMe(22:5(4Z,7Z,10Z,13Z,16Z)/22:6(4Z,7Z,10Z,13Z,16Z,19Z)) | 1.317 | 0.042 | 0.947 | Down |
| Tetradecanedioic acid | 1.653 | 0.038 | 0.942 | Down |
| Hydroxy-alpha-sanshool | 1.357 | 0.035 | 0.936 | Down |
| Panaxydol linoleate | 1.844 | 0.004 | 0.923 | Down |
| Annosquamosin B | 1.813 | 0.006 | 0.922 | Down |
| (9Z,11R,12S,13S,15Z)-12,13-^4^ | 2.200 | 0.008 | 0.880 | Down |
| Ethyl 2-hydroxy-3-(3-indolyl)propanoate glucoside | 2.377 | 0.042 | 0.749 | Down |
| Glycinoeclepin A | 2.850 | 0.010 | 0.729 | Down |
| 6b-Angeloyl-3b,8b,9b-trihydroxy-7(11)-eremophilen-12,8-olide | 3.483 | 0.011 | 0.584 | Down |
| Taurochenodeoxycholate-3-sulfate | 4.137 | 0.010 | 0.553 | Down |
| 10,20-Dihydroxyeicosanoic acid | 5.489 | 0.004 | 0.460 | Down |
| Taurochenodeoxycholate-7-sulfate | 4.281 | 0.002 | 0.346 | Down |
| Organic acids and derivatives (7) | | | | |
| Asparaginyl-Alanine | 2.860 | 0.009 | 1.699 | Up |
| D-Pipecolic acid | 2.276 | 0.028 | 1.280 | Up |
| Alanyl-Arginine | 1.387 | 0.048 | 1.064 | Up |
| Fumonisin B3 | 1.114 | 0.009 | 1.026 | Up |
| L-Histidine | 1.081 | 0.021 | 0.959 | Down |
| Isoputreanine | 1.805 | 0.016 | 0.901 | Down |
| L-Proline | 2.579 | 0.000 | 0.818 | Down |
| Organic oxygen compounds (4) | | | | |
| Tyramine glucuronide | 2.962 | 0.002 | 1.287 | Up |
| (-)-erythro-Anethole glycol 1-glucoside | 1.074 | 0.037 | 0.955 | Down |
| De-O-methylsimmondsin | 1.675 | 0.024 | 0.917 | Down |
| Galactose-beta-1,4-xylose | 1.854 | 0.016 | 0.899 | Down |
| Benzenoids (3) | | | | |
| 2-[(4-methoxyphenyl)formamido]acetic acid | 1.898 | 0.039 | 1.184 | Up |
| P-Hydroxyphenylacetic acid | 1.941 | 0.039 | 1.142 | Up |
| 4'-Hydroxyfenoprofen glucuronide | 3.498 | 0.003 | 0.437 | Down |
| Phenylpropanoids and polyketides (3) | | | | |
| (3-oxo-1,3-diphenylpropoxy)sulfonic acid | 1.358 | 0.043 | 1.113 | Up |
| Ganodermic acid TQ | 1.202 | 0.030 | 0.945 | Down |
| Hordatine A | 2.322 | 0.034 | 0.694 | Down |
| Nucleosides, nucleotides, and analogues (2) | | | | |
| Diadenosine tetraphosphate | 1.522 | 0.043 | 0.897 | Down |
| Guanosine | 2.507 | 0.024 | 0.838 | Down |
| Organoheterocyclic compounds (1) | | | | |
| Indoleacetic acid | 2.309 | 0.039 | 1.219 | Up |
| Others (4) | | | | |
| 1-Amino-1-cyclopentanecarboxylic acid | 1.944 | 0.000 | 0.919 | Down |
| 3-Indolepropionic acid | 2.161 | 0.005 | 0.885 | Down |
| 2-Aminomethylpyrimidine (hydrochloride) | 1.569 | 0.007 | 0.870 | Down |
| C16 Sphinganine | 3.525 | 0.001 | 0.829 | Down |

^1^VIP = VIP_OPLS-DA.

^2^FC = fold change.

^3^“up” means the serum compound had a greater concentration in follicular cyst ewes as compared with normal follicle ewes.

^4^(9Z,11R,12S,13S,15Z)-12,13- = (9Z,11R,12S,13S,15Z)-12,13-Epoxy-11-hydroxy-9,15-octadecadienoic acid.
